# Benign Scrotal Tumor in a Pediatric Patient: Epididymal Cyst

**DOI:** 10.1155/2018/1635635

**Published:** 2018-06-20

**Authors:** María Fernández-Ibieta, Flor Villalon-Ferrero, Jose Luis Ramos-García

**Affiliations:** ^1^Pediatric Surgery, Hospital Clínico Universitario Virgen de la Arrixaca, Murcia, Spain; ^2^Pediatric Surgery, Hospital Universitario Donostia, San Sebastian, Spain

## Abstract

A pediatric patient of 12 years consulted for a left scrotal mass of 2 months of evolution. After suspecting a cystic content due to positive transillumination, on ultrasonography a scrotal cyst separated from the testis, of 5 cm in its maximum length, was confirmed. Due to size, parental anxiety, and the referred short evolution, excision was decided. Given the clinical radiological findings, a scrotal incision was chosen, obtaining complete excision. Biopsy confirmed the diagnosis of simple epididymal cyst (EC). ECs usually present as painless, scrotal swelling in adolescents as a result of dilatation of the efferent epididymal tubules. Many cases (up to 60%) regress spontaneously. In these, average time to involute ranges from 4 to 50 months. Although cases of cyst torsion have been described (with pain derived from ischemia and inflammation), conservative management has been suggested in the majority, both in pediatric and in adult series. Surgery is recommended in some patients, due to testicular pain or increased paratesticular mass, as was our case.

## 1. Introduction

The most common extratesticular lesions encountered in children include Morgagni hydatid torsion, epididymitis, paratesticular rhabdomyosarcoma, epididymal cysts/spermatoceles, and varicocele. As opposed to intratesticular masses, most extratesticular masses are benign. Examples of rare extratesticular lesions in adults include lipomas (most often arising from the spermatic cord), adenomatoid tumors (most often found in the epididymis), sarcoidosis, liposarcoma, leiomyosarcoma, malignant fibrous histiocytoma, mesothelioma, and lymphoma [1–3].

## 2. Case Report

**Figure 1:**
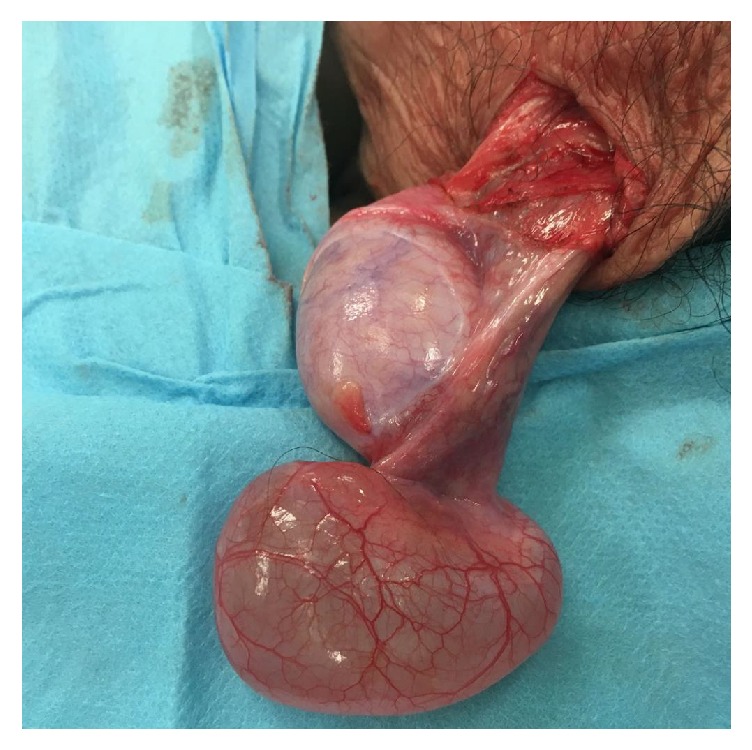
Surgical image of EC adjacent to the left testis.

**Figure 2:**
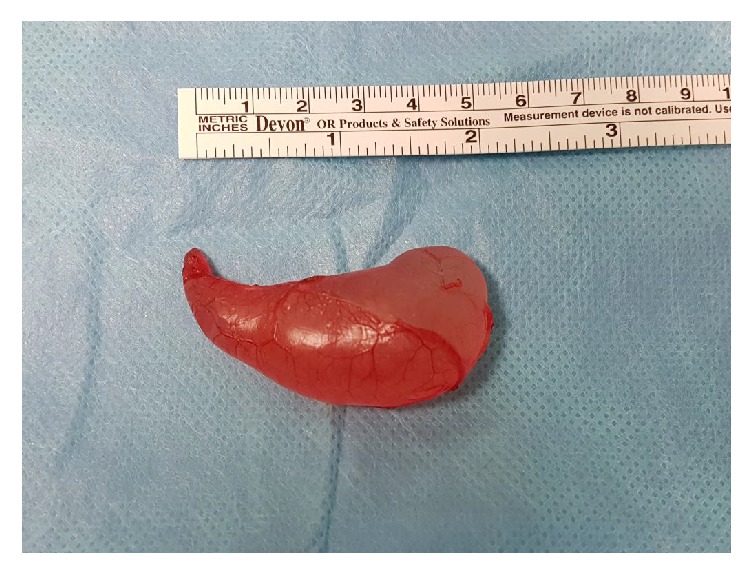
The cyst, once excised.

A pediatric patient of 12 years consulted for a left scrotal mass of 2 months of evolution. It was not painful, and he did not present any other associated symptoms. On examination, testes appeared normal in size and location, but a 4 cm oval soft scrotal mass was palpated in the left scrotum, adjacent to the testis. It was of elastic consistency, mobile, not adhered, and separated from the left testis. Given the suspicion of epididymal cyst (EC), after positive transillumination, ultrasound was requested. An uncomplicated scrotal cyst separated from the testis, of 5 cm in its maximum length, was confirmed. Due to the size, parental anxiety, and the referred short evolution, preferred excision was decided. Given the clinical radiological findings, where a malign tumor was not suspected, a scrotal incision was chosen, obtaining complete excision of the cyst, adjacent to the epididymis head. Figures 1 and 2. Pathological anatomy confirmed the diagnosis of simple EC. Evolution was uneventful without recurrence in the following year.

## 3. Discussion

EC usually presents as painless, scrotal swelling in adolescents as a result of dilatation of the efferent epididymal tubules. On many occasions terms ‘EC' and ‘spermatocele' have been used interchangeably to describe the same entity. The only means of differentiating these 2 lesions is aspiration of the cyst content, as EC does not contain sperm [3, 4]. Histologically, spermatoceles contain thick fluid with nonviable spermatozoa and debris. Sonographically, they are seen as thin-walled, septated cysts within the epididymal head [3] with dependent echoes. EC occurs at any age, can be found anywhere along the epididymis, does not contain spermatozoa, and therefore appears more simple sonographically [1]. Up to 20% of the cases can be bilateral.

Previous reports revealed EC in 5% of pediatric patients undergoing scrotal ultrasound and in 15% of boys undergoing ultrasound for a palpable mass [5]. Some authors refer to an increase in the proportion of EC with age [5] as they usually develop around the age of 40 [3], and in another recent work [6], more than 30% of adult men, fertile or infertile, show cysts on sonography. There is confusing data regarding prevalence (14-50%), after all, depending on sample characteristics (prepubertal boys or adult men who seek fertility)

Etiology of EC remains unknown. The result of endocrine disrupting agents acting fetally or postnatally may play a role in EC development, and obstruction to the flow of sperm content has also been described. Epididymal epithelium is dependent on a relatively high concentration of androgens for normal function. This dependence includes secretory activity, fluid resorption, and cytological integrity. Recently, an apparent increase in the number of cases has been pointed out by some authors, theoretically caused by increasing exposure to estrogenic compounds in the environment [4]. On the other hand, a pathological report described epididymal cyst as a structure that originates from vestigial remnants of epididymis that is not communicating with epididymal tubules. Whether these remnants are mesonephric or müllerian in origin is not known. ECs have also been associated with Cystic Fibrosis and von Hippel Lindau syndrome [3, 4].

Pain and scrotal mass are the most common clinical findings in patients with EC.

Many cases (up to 60%) regress spontaneously [3, 4]. In these, average time to complete involution ranges from 4 to 50 months [3, 4]. Size increase has not been related to any particular risk yet. Although cases of cyst torsion have been described [7](with pain derived from ischemia and inflammation), conservative management, through periodic ultrasound follow-up, has been suggested in the majority, both in pediatric and in adult series. Surgery is recommended in some patients, due to nonmanageable testicular pain or increased paratesticular mass, as it was our case. The approach can be through scrotal skin if no testicular tumor is suspected [8]. Cyst size may play a role in deciding the choice of treatment. Conservative management of ECs smaller than 10 mm has been suggested while leaving surgery for cysts over 10 mm in diameter [3]. Although one series [4] reported the risk of recurrence after surgery, others have not shown such an effect.

Our case illustrates a rare specimen of EC of a considerable size in a preadolescent male, showing the ease to perform the excision through a scrotal incision.
